# Supplementary material for: The interactome of intact mitochondria by cross-linking mass spectrometry provides evidence for coexisting respiratory supercomplexes
Source: Mol Cell Proteomics. 2017 Dec 8;17(2):216–32. doi: 10.1074/mcp.RA117.000470 (PMC5795388; doi:10.1074/mcp.RA117.000470)
Supplement: Supplemental Data [file supp_RA117.000470_133922_0_supp_23978_fzffwf.zip › spectra_annotation/mito_spectra_annotation/149-1-18-1-6-1.pdf]

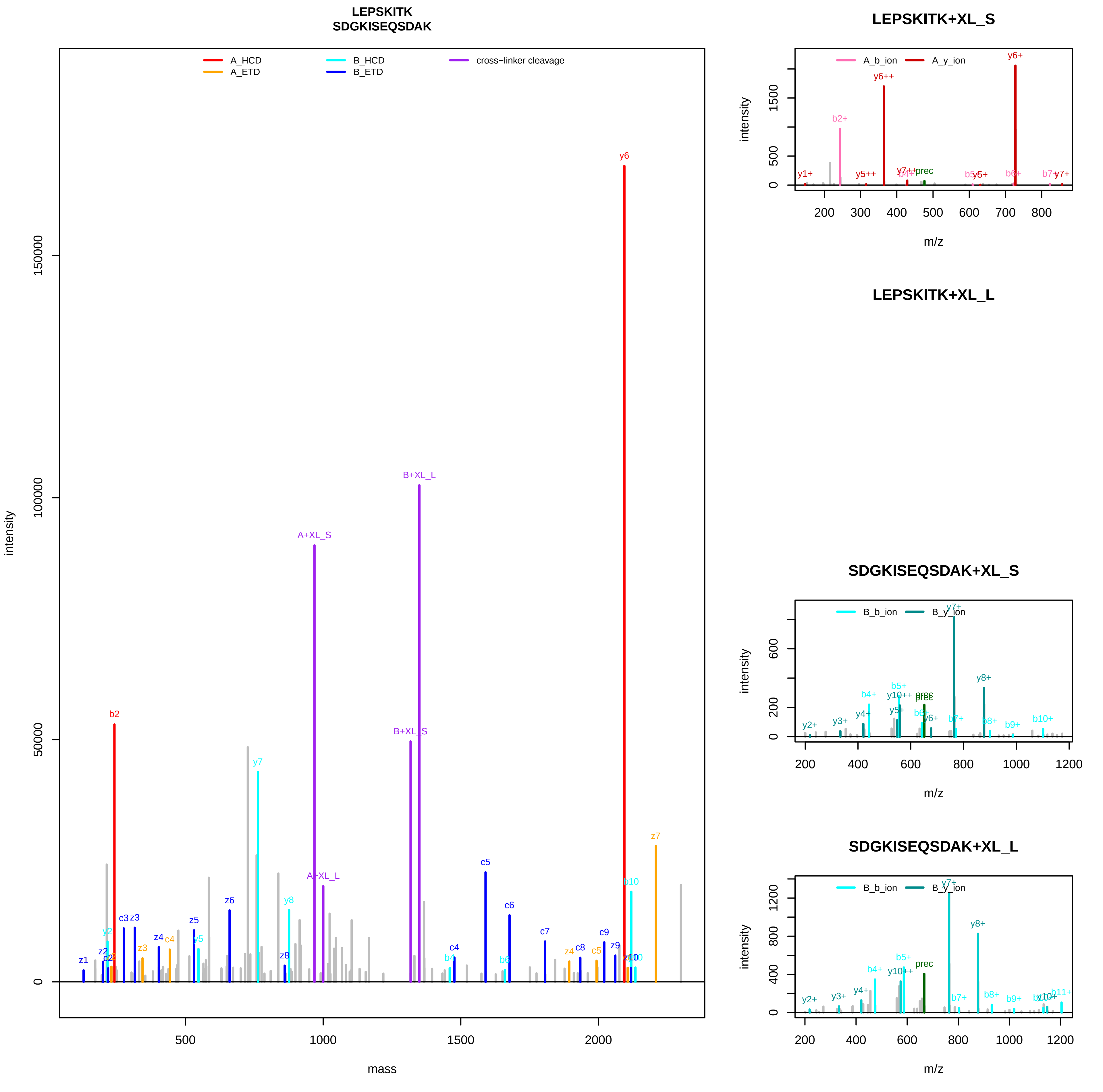

LEPSKITK
SDGKISEQSDAK
A_HCD
A_ETD
B_HCD
B_ETD
cross−linker cleavage
intensity
mass
0
50000
100000
150000
500
1000
1500
2000
y6
B+XL_L
A+XL_S
B+XL_S
b2
y7
z7
A+XL_L
c5
b10
z6
y8
c6
c3
z3
z5
c7
c9
z9
z4
c4
z3
y2
y5
c4
c8
z4
c5
z10
z2
c2
z1
z8
b4
b6
LEPSKITK+XL_S
A_b_ion
A_y_ion
y6+
y6++
b2+
y1+
y5++
y7++
prec
b4+
b5+
y5+
b6+
b7+
y7+
m/z
200
300
400
500
600
700
800
0
500
1500
LEPSKITK+XL_L
SDGKISEQSDAK+XL_S
B_b_ion
B_y_ion
y7+
y8+
b4+
b5+
y10++
prec
y5+
b6+
y6+
b7+
b8+
b9+
b10+
y2+
y3+
y4+
0
200
600
1000
1200
SDGKISEQSDAK+XL_L
y7+
y8+
b5+
b4+
y10++
prec
y4+
y3+
y2+
b7+
b8+
b9+
y10+
b11+
0
400
800
1200
